# ADULT DAY HEALTH SERVICES ATTENDANCE IS LINKED TO A REDUCTION IN LONELINESS

**DOI:** 10.1093/geroni/igz038.3502

**Published:** 2019-11-08

**Authors:** Wingyun Mak, Orah R Burack, Joann P Reinhardt

**Affiliations:** The New Jewish Home, New York, New York, United States

## Abstract

Loneliness is the subjective experience of inadequate social contact, and it is linked to numerous detrimental psychological and medical outcomes like depression, cognitive decline, sleep fragmentation, metabolic syndrome, diminished immune functioning, and morbidity. Older adults with cognitive impairment and/or other comorbidities are at greater risk for loneliness due to diminishing social roles, functional status, and death of peers. They are often encouraged to attend adult day services to engage in an environment of socialization and supported activities, and in the case of adult day health services, additional medical services such as physical therapy, skilled nursing care, medical management, and nutritional counseling. In this study we examined whether attending adult day health services (ADHS) at least once a week would be associated with decreased levels of loneliness across time. Our data came from a sample of older adults attending ADHS in New York City from 2018-2019 who scored five or greater on the Nursing Facility Level of Care Index, which is a score derived from assessments of cognition, communication and vision, mood and behavior, functional status, continence, and nutritional status from the Uniform Assessment System in New York (UAS-NY). We found that attendance was linked with fewer reports of loneliness across time, ∑2(1, N=563) = 21.33, p<.001. These results highlight the importance of attending adult day health services for people with a complex health status and the potential role ADHS may play in reducing loneliness in a vulnerable population.

